# The Outcomes of SJS/TEN: A Nationwide Analysis

**DOI:** 10.1093/jbcr/irag010

**Published:** 2026-01-29

**Authors:** Tyler J Murphy, Arman J Fijany, Emily P Swafford, Jordan T Garcia, Punit Vyas, Robel T Beyene, Stephen P Gondek, Anne L Wagner, Mayur B Patel, Elizabeth Dale Slater

**Affiliations:** Department of Surgery, Vanderbilt University Medical Center, 1310 24th Avenue South, Nashville, TN 37212, United States; Critical Illness, Brain dysfunction, and Survivorship (CIBS) Center, Center for Health Services Research, Vanderbilt University Medical Center, 2525 West End Avenue, Nashville, TN 37212, United States; Department of Surgery, Vanderbilt University Medical Center, 1310 24th Avenue South, Nashville, TN 37212, United States; Department of Plastics and Reconstructive Surgery, University of California – Davis Health, 4301 X Street, Sacramento, CA 95817, United States; Department of Surgery, Vanderbilt University Medical Center, 1310 24th Avenue South, Nashville, TN 37212, United States; Department of Surgery, Vanderbilt University Medical Center, 1310 24th Avenue South, Nashville, TN 37212, United States; Department of Surgery, Vanderbilt University Medical Center, 1310 24th Avenue South, Nashville, TN 37212, United States; Department of Surgery, Vanderbilt University Medical Center, 1310 24th Avenue South, Nashville, TN 37212, United States; Department of Surgery, Vanderbilt University Medical Center, 1310 24th Avenue South, Nashville, TN 37212, United States; Department of Surgery, Vanderbilt University Medical Center, 1310 24th Avenue South, Nashville, TN 37212, United States; Department of Surgery, Vanderbilt University Medical Center, 1310 24th Avenue South, Nashville, TN 37212, United States; Critical Illness, Brain dysfunction, and Survivorship (CIBS) Center, Center for Health Services Research, Vanderbilt University Medical Center, 2525 West End Avenue, Nashville, TN 37212, United States; Department of Surgery, Vanderbilt University Medical Center, 1310 24th Avenue South, Nashville, TN 37212, United States

**Keywords:** stevens-Johnson syndrome, toxic epidermal necrolysis syndrome, outcomes, burn surgery, respiratory failure

## Abstract

Stevens–Johnson syndrome (SJS) and toxic epidermal necrolysis (TEN) syndrome are potentially fatal skin conditions frequently cared for in burn units. In a national database study, we studied the demographics and outcomes of this patient population. This retrospective cohort study included patient admissions for SJS/TEN. Patient demographics and outcomes were compared and adjusted for age, sex, inhalation injury, and percent total body surface area (TBSA). Logistical regression was used for binary outcomes, and linear regression was used for continuous outcomes. All outcomes were described in reference to the entire disease continuum (SJS/TEN) and each specific disease subcohort (SJS, SJS-TEN overlap, and TEN). Of 271 971 patients queried within the Noncommercial Burn Research Dataset, 2416 patients had a diagnosis of SJS/TEN. These patients were statistically more likely to be older (48 ± 22 years vs 36 ± 22 years), housed (1% vs 2%), females (57% vs 34%), and have a higher mean TBSA (8 ± 13% vs 7 ± 12%). In multivariable analysis, SJS/TEN was associated with increased risk of unplanned intubations (odds ratio [OR] 1.69) and pneumonia (OR 1.26), but not respiratory failure (OR 0.36). There was also an increased risk for sepsis (OR 1.43). Patients were significantly more likely to have a shorter hospital LOS (OR −3.7). There was no significant difference in mortality. In subcohort analysis, a stepwise increase in morbidity and mortality was observed when comparing SJS, SJS-TEN overlap, and TEN. Stevens–Johnson syndrome/TEN is a rare but extremely morbid disease continuum that frequently affects female patients and results in increased respiratory and alternative complications.

## INTRODUCTION

First described by Lyell in 1956 as a pathology that was distinctly different from staphylococcal scalded skin syndrome, Stevens–Johnson syndrome (SJS), and toxic epidermal necrolysis (TEN) syndrome are a continuum of rare and severe cutaneous disorders which are defined by widespread necrosis of the skin and mucosa.^1–4^ The disease continuum of SJS/TEN is defined by the cutoffs of less than 10% total body surface area (TBSA) for SJS, 10%-30% TBSA involvement described as SJS-TEN overlap, and more than 30% TBSA termed TEN.^5^ Stevens–Johnson syndrome/TEN has been cited to occur in around 9.2, 1.6, and 1.9 cases per million person years for SJS, SJS-TEN overlap, and TEN, respectively, with mortality rates of 10%, 30%, and 50%.^2^^,^^3^^,^^6^

In smaller cohort studies, it has been stated that SJS/TEN occurs more frequently in elderly, immunocompromised male patients with comorbidities such as cancer, type 2 diabetes mellitus, and chronic liver disease.^6–9^ Frequently these at risk individuals consume certain medications, cited to be the cause for SJS/TEN in upward of 85% of cases, or are exposed to infectious pathogens as the inciting event for their skin reaction.^4^^,^^7^^,^^10^ Particularly the medication classes of antibiotics (eg, doxycycline, ciprofloxacin), anticonvulsants (eg, nevirapine, lamotrigine), and allopurinol are frequently implicated.^11^ The mechanism of injury is cited to relate to T-cell-mediated type IV hypersensitivity reaction to these agents leading to widespread keratinocyte death.^4^^,^^12^ Mortality is frequently attributed to both acute and long-term complications including sepsis, multisystem organ failure, urogenital scarring, and cardiopulmonary complications as a direct result of the mucosal/skin necrosis seen in the pathology.^2^^,^^3^^,^^6^^,^^10^^,^^13–19^ Currently, treatment pertains to treating or stopping the causative agent and symptom management with inconsistency as to what is done for wound care or systemic therapy.^4^^,^^18^^,^^20^

This manuscript aims to provide an in-depth national epidemiologic analysis of the patient population afflicted by the SJS/TEN disease continuum, with individualized analysis of each subcohort, and their outcomes when compared to their patient counterparts with burn injuries also cared for in tertiary burn centers, with the hypothesis that they will be at greater risk for respiratory complications requiring intervention, on a population scale that has not been previously described using the American Burn Association (ABA) Noncommercial Burn Research Dataset (NBRD).

## MATERIALS AND METHODS

The ABA NBRD was queried for patients with burn injuries who were cared for with the diagnosis of SJS, SJS-TEN overlap, or TEN per ICD10 diagnosis codes from 2012 to 2021 in a participating burn center.^21^ The ABA NBRD consists of patients cared for in 109 nationwide burn centers from 39 states in addition to Washington, DC and is upkept by the ABA with annual updates and audits.^21^ Two major cohorts of patient admissions were isolated by unique patient ICD10 code—those with a diagnosis of SJS, SJS-TEN overlap, or TEN (either primary or secondary diagnoses) and patients with burn injuries cared for in a burn center without these diagnoses. Within these 2 major cohorts, demographics, clinical outcomes, complications, and injury characteristics were delineated.

The primary outcomes of interest were pneumonia, unplanned intubations, and respiratory failure as described by the ABA NBRD.^21^ We further investigated the secondary outcomes that included mortality, intensive care unit (ICU) admission, systemic sepsis, renal failure, multiple organ failure, and hospital length of stay (LOS). Each outcome was studied within the total cohort (SJS/TEN) and each subcohort of disease (SJS, SJS-TEN overlap, and TEN). To ensure accurate stratification, patients with SJS/TEN having unknown TBSA involvement were excluded from subcohort analyses.

Multivariable regression analyses were performed for all primary and secondary outcomes, with associated covariate adjustments for the variables of age, sex, TBSA percentage, and inhalation injury. Ordinary least squares (OLS) regression was used to describe hospital LOS. Patients without data for outcomes analyzed, or any of the 4 major covariates were excluded from the regression analyses. Categorical variables were converted into binary values (eg, presence of sepsis = 1, no sepsis = 0). Continuous variables included age and TBSA%. Age was assessed by 1-year increments for effects on outcomes, whereas TBSA% data were divided into bins of 10% increments. The first category, 0%-10%, comprised the reference category for comparison in the regression model. No imputation was performed; missing covariates were handled via complete-case analysis. Data were inspected for impossible or non-numeric values and cleaned before modeling.

All primary and secondary outcome results derived from multivariate regression were reported with odds ratios (ORs) with 95% confidence intervals (CIs). For LOS, the OLS regression reported results using the β coefficient with a 95% CI. Each analysis was performed for the total cohort (SJS/TEN) and each subcohort of disease (SJS, SJS-TEN overlap, and TEN). All statistical analysis was performed in PyCharm 3.1 software using pandas, NumPy, and SciPy.Stats modules, with significance set as *P* < .05.

## RESULTS

Of 271 971 patients with burn injuries queried, 2416 patients had an ICD10 code diagnosis for SJS, SJS-TEN overlap, or TEN showing a 0.9% prevalence among patients cared for in burn centers throughout the United States. Baseline demographics of this study are found in Table 1. Patients with SJS/TEN were more likely to be female ([% SJS/TEN vs % non-SJS/TEN; *P*-value]; 57.0% vs 34.0%; *P* < .001). Patients suffering from SJS/TEN were more likely to be Black (29.0% vs 20.0%; *P* < .001) or Asian (5.0% vs 2.0%; *P* < .001) race while patients with non-SJS/TEN were more likely to be White (51.0% vs 57.0%; *P* < .001). Patients with SJS/TEN were less likely to be homeless (1.0% vs 2.0%; *P* < .001). Patients with SJS/TEN were more likely to be older with an average age of 48 years (SD 22.0) vs patients with non-SJS/TEN burn injuries having an average age of 36 years of age (SD 24.0). Of note, the prevalence of inhalation injury was lower in patients with SJS/TEN when compared to their counterparts (0.0% vs 7.0%; *P* < .001). Finally, the average TBSA was larger in SJS/TEN when compared to patients with non-SJS/TEN (8.0% vs 7.5%; *P* < .001).

**Table 1:** Study Population Demographics

	SJS/TEN (*n* = 2416)	Non-SJS/TEN (*n* = 269 555)
**Sex** ^**a**^		
**Male**	1 047 (43%)	179 187 (66%)
**Female**	1 369 (57%)	90 368 (34%)
**Race** ^**a**^		
**White**	1 244 (51%)	154 852 (57%)
**Black**	698 (29%)	54 398 (20%)
**Asian**	127 (5%)	6 134 (2%)
**American Indian/Alaska Native**	17 (1%)	2 204 (1%)
**Homeless** ^**a**^	15 (1%)	4 308 (2%)
**Age (years, SD)** ^**a**^	48 (22)	36 (24)
**Inhalation injury** ^**a**^	1 (0%)	19 528 (7%)
**Mean TBSA (%, SD)** ^**a**^	8.02% (13.37)	7.49% (12.25)

Abbreviations: SJS, Stevens–Johnson syndrome; TEN, toxic epidermal necrolysis.

Baseline demographics at presentation for patients with SJS/TEN and non-SJS/TEN burn injuries. All values are number of individuals (percent total population) unless otherwise specified.

a Denotes significance.

**Figure 1:**
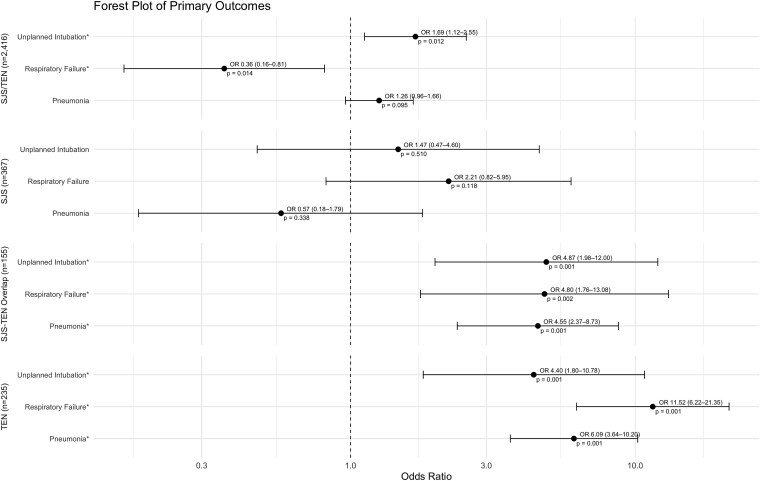
Patient Outcomes—Respiratory. Respiratory Outcomes for SJS/TEN, SJS, SJS-TEN Overlap, and TEN. Abbreviations: SJS = Stevens–Johnson syndrome; TEN = toxic epidermal necrolysis. ^*^ denotes significance.

Patients who were diagnosed with SJS/TEN were at significantly increased risk for unplanned intubations (OR 1.69, *P* = .012; Table 2, Figure 1) with a numerically increased risk for pneumonia that was not statistically significant (OR 1.26, *P* = .095). In this national cohort, there was a decreased risk in patients with SJS/TEN for respiratory failure (OR 0.36, *P* = .014) when compared to the population of control patients with burn injuries. Patients with SJS did not show a statistically significant increased risk of unplanned intubations (OR 1.47, *P* = .510) or respiratory failure (2.21, *P* = .118). Those patients with SJS-TEN overlap and TEN showed a significantly increased risk of pneumonia, unplanned intubations, and respiratory failure. Patients diagnosed with SJS-TEN overlap showed a significantly increased risk of pneumonia (OR 4.55, *P* < .001), unplanned intubations (OR 4.87, *P* < .001), and respiratory failure (OR 4.80, *P* = .002). Those patients with TEN showed a statistically elevated odds of experiencing pneumonia (OR 6.09, *P* < .001), unplanned intubations (OR 4.40, *P* = .001), and respiratory failure (OR 11.52, *P* < .001) during their admission.

**Table 2:** Patient Outcomes—Respiratory

*Primary outcomes SJS/TEN* *(n = 2416)*	OR	CI	*P*-value
**Pneumonia**	1.26	0.96-1.66	.095
**Unplanned intubation** ^**a**^	1.69	1.12-2.55	.012
**Respiratory failure** ^**a**^	0.36	0.16-0.81	.014
*Primary outcomes SJS* *(n = 367)*	**OR**	**CI**	***P*-value**
**Pneumonia**	0.57	0.18-1.79	.338
**Unplanned intubation**	1.47	0.47-4.60	.510
**Respiratory failure**	2.21	0.82-5.95	.118
*Primary outcomes SJS-TEN overlap* *(n = 155)*	**OR**	**CI**	***P*-value**
**Pneumonia** ^**a**^	4.55	2.37-8.73	<.001
**Unplanned intubation** ^**a**^	4.87	1.98-12.00	<.001
**Respiratory failure** ^**a**^	4.80	1.76-13.08	.002
*Primary outcomes TEN* *(n = 235)*	**OR**	**CI**	***P*-value**
**Pneumonia** ^**a**^	6.09	3.64-10.20	<.001
**Unplanned intubation** ^**a**^	4.40	1.80-10.78	.001
**Respiratory failure** ^**a**^	11.52	6.22-21.35	<.001

Abbreviations: SJS, Stevens–Johnson syndrome; TEN, toxic epidermal necrolysis.

Respiratory outcomes for patients with SJS/TEN and non-SJS/TEN burn injuries. All values are presented as odds ratio (OR) with respective confidence intervals (CIs).

a Denotes statistical significance.

**Figure 2:**
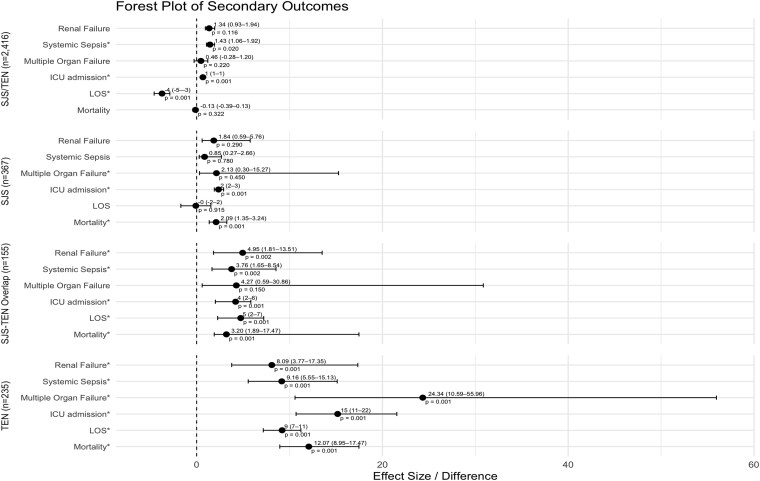
Patient Outcomes—General. General Outcomes for SJS/TEN, SJS, SJS-TEN Overlap, and TEN. Abbreviations: ICU = intensive care unit; LOS = length of stay; SJS = Stevens–Johnson syndrome; TEN = toxic epidermal necrolysis. ^*^ denotes significance.

In secondary analysis, it was seen that patients with SJS/TEN were less likely to require ICU admissions (OR 0.67, *P* < .001; Table 3, Figure 2) and had a shorter overall LOS (OR −3.73, *P* < .001). There was no significant difference in mortality between the 2 groups (OR −0.13, *P* = .322). Patients with SJS/TEN were found to be at an increased risk for renal failure (OR 1.34, *P* = .116) and less likely to exhibit multiorgan failure (OR 0.46, *P* = .22), albeit neither were statistically significant. Furthermore, patients were at an increased risk for developing systemic sepsis with an OR of 1.43 (*P* =. 02) when compared to patients with non-SJS/TEN burn injuries.

**Table 3:** Patient Outcomes—General

*Secondary outcomes SJS/TEN* *(n = 2416)*	OR	CI	*P*-value
**Renal failure**	1.34	0.93-1.94	.116
**Systemic sepsis** ^**a**^	1.43	1.06-1.92	.02
**Multiple organ failure**	0.46	−0.28-1.20	.22
**ICU admission** ^**a**^	0.67	0.53-0.82	<.001
**LOS** ^**a**^	−3.73	−4.58-−2.89	<.001
**Mortality**	−0.13	−0.39-0.13	.322
*Secondary outcomes SJS* *(n = 367)*	**OR**	**CI**	***P*-value**
**Renal failure**	1.84	0.59-5.76	.29
**Systemic sepsis**	0.85	0.27-2.66	.78
**Multiple organ failure** ^**a**^	2.13	0.30-15.27	.45
**ICU admission** ^**a**^	2.35	1.90-2.90	<.001
**LOS**	−0.09	−1.705-1.529	.9153
**Mortality** ^**a**^	2.09	1.35-3.24	.001
*Secondary outcomes SJS-TEN overlap* *(n = 155)*	**OR**	**CI**	***P*-value**
**Renal failure** ^**a**^	4.95	1.81-13.51	.002
**Systemic sepsis** ^**a**^	3.76	1.65-8.54	.002
**Multiple organ failure**	4.27	0.59-30.86	.15
**ICU admission** ^**a**^	4.19	2.01-5.83	<.001
**LOS** ^**a**^	4.74	2.26-7.22	<.001
**Mortality** ^**a**^	3.20	1.89-17.47	<.001
*Secondary outcomes TEN* *(n = 235)*	**OR**	**CI**	***P*-value**
**Renal failure** ^**a**^	8.09	3.77-17.35	<.001
**Systemic sepsis** ^**a**^	9.16	5.55-15.13	<.001
**Multiple organ failure** ^**a**^	24.34	10.59-55.96	<.001
**ICU admission** ^**a**^	15.18	10.70-21.55	<.001
**LOS** ^**a**^	9.20	7.17-11.22	<.001
**Mortality** ^**a**^	12.07	8.95-17.47	<.001

Abbreviations: ICU, intensive care unit; LOS, length of stay; SJS, Stevens–Johnson syndrome; TEN, toxic epidermal necrolysis.

General outcomes for patients with SJS/TEN and non-SJS/TEN burn injuries. All values are presented as odds ratio (OR) with respective confidence intervals (CIs).

a Denotes statistical significance.

Within subcohort analyses, patients diagnosed with SJS had a statistically significant increased risk of mortality (OR 2.09, *P* = .001) and ICU admission (OR 2.35, *P* < .001). There was no statistical difference in patients with SJS for the outcomes of renal failure, multiple organ failure, systemic sepsis, or LOS when compared to patients with non-SJS/TEN burn injuries. Patients with SJS-TEN overlap had an increased risk of renal failure (OR 4.95, *P* = .002), systemic sepsis (OR 3.76, *P* = .002), ICU admission (OR 4.19, *P* < .001), and mortality (OR 3.2, *P* < .001). In addition, there was an increase in LOS by almost 5 days (OR 4.74, *P* < .001). There was an increased OR of 4.27 in relation to multiple organ failure within the SJS-TEN overlap population, but this was not statistically significant (*P* = .15). Within the TEN subcohort analyses, patients were more likely to experience renal failure (OR 8.09, *P* <. 001), systemic sepsis (OR 9.16, *P* < .001), multiple organ failure (OR 24.34, *P* < .001), ICU admission (OR 15.18, *P* < .001), and mortality (OR 12.07, *P* < .001). These patients experienced a greater LOS by 9 days (OR 9.2, *P* < .001).

## DISCUSSION

The findings within this study provide a multi-institutional analysis of patient demographics and respiratory and general outcomes in patients with SJS/TEN cared for in burn centers when compared to their patient counterparts with burn injuries. Our findings show SJS/TEN more frequently affects older, housed females when compared to their patient counterparts with burn injuries. We demonstrated that patients with SJS/TEN are more likely to undergo unplanned intubation and develop pneumonia but did not have increased odds of respiratory failure when compared to the burn population. As patients progress on the disease continuum toward TEN, the odds of experiencing adverse respiratory outcomes increased. In addition, known associations of SJS/TEN and urogenital involvement and systemic sepsis were found, however, an increased risk of multiorgan failure when compared to patients with burn injuries was not supported in the general SJS/TEN population, but was identified in the TEN group. Our averaged findings suggest that patients with SJS/TEN are less likely to need an ICU and have shorter LOS than their patient counterparts with burn injuries having no significant difference in mortality, but the subgroup analysis demonstrated these outcomes were more likely to be experienced as patients increased in acuity from SJS to TEN.

In contrast to previous studies, our findings show that SJS/TEN more frequently affects women than men when compared to their patient counterparts with burn injuries.^3^^,^^22^ It has been described that this may be due to males having suppressed delayed-type hypersensitivity reactions like those seen in SJS/TEN.^23^ Another reason for this discrepancy could be due to the overwhelming prevalence of male involvement in accidental burn injury.^24^ However, this is an important concept to include in SJS/TEN when looking at unique outcomes for women within the disease process. Specifically, it has been cited that upward of 77% of women diagnosed with SJS/TEN have gynecological involvement.^14^^,^^25^ Menstrual disturbances as well as vulvar and vaginal adenosis, vaginal stenosis, fusion of labia minora or majora, and possible complete vaginal fusion following SJS/TEN can lead to obstruction of menstrual blood flow causing long-term abdominal pain, dyspareunia, hematocolpos, and hydrocolpos.^25^^,^^26^ In response to the increased risk of gynecological complications, some burn centers have made gynecology consultation as part of their protocol for female patients early in the care of SJS/TEN.^14^ This finding brings to the forefront of discussion that is important for the clinician to be educated and mindful of the outcomes unique to women following SJS/TEN.

Respiratory complications secondary to mucosal sloughing can lead to multiple adverse outcomes both in the acute and chronic setting.^26^ In the acute setting, it has been cited that upward of 40% of patients with SJS/TEN have respiratory involvement and 38% may require mechanical ventilation.^3^^,^^14^^,^^25^^,^^27^ Our data show a significantly increased risk for unplanned intubations as well as an increased risk of pneumonia occurrence within patients with SJS/TEN when compared to patients with burn injuries when controlling for inhalation injury. However, we did not find an increased odds of respiratory failure when compared to their patient counterparts with burn injuries in the general population. As expected, these complications are more likely to occur as TBSA involvement increases transitioning from SJS to TEN. Previously, criterion has been suggested that those patients with SJS/TEN having oral mucosa involvement in addition to one of the following clinical features: initial TBSA involvement > 70%, progression of TBSA since admission > 15%, underlying neurological diagnosis inhibiting protection of airway, and visualization of airway involvement with direct laryngoscopy; should be prophylactically intubated to improve outcomes.^28^ Beyond this, there are currently no guidelines to help with ventilatory management or specific pneumonia treatment in the SJS/TEN population in the setting of their unique clinical vulnerability from the mucosal sloughing of the respiratory tract. This is a major gap in SJS/TEN care as it has been shown that the effects of these acute complications are not short-term with abnormal pulmonary function tests showing moderate impairment in alveolar dysfunction upwards of 2 months postdischarge.^29^ In the setting of known respiratory involvement and without current guidelines, there is a need for further development of intubation criterion development, pneumonia treatment, and ventilatory management strategies to best identify and treat those at risk for respiratory complications.

Other short-term complications seen within our study included systemic sepsis and renal failure. As expected, these complications are more likely to occur as TBSA involvement increases transitioning from SJS to TEN. Sepsis frequently occurs from the lack of protective coverage after mucosal and skin sloughing leading to increased risk of nosocomial infections. Currently, there are no universally accepted clinical practice for wound management or antibiotic stewardship in SJS/TEN.^13^ Cited within our study is the increased odds toward renal failure in patients with SJS/TEN observed within the SJS-TEN overlap and TEN subcohorts. This is likely due to our control group, general patients with burn injuries, similarly having increased risk of renal failure being cited upward of 30% for patients with > 10% TBSA burns.^30^ Comparatively within the SJS/TEN population, it has been shown that acute kidney injury is present in upward of 18.8% of all cases with around 5.0% of those going on to require long-term dialysis.^3^^,^^16^ This is likely secondary to both prerenal kidney injury from fluid shifts and direct kidney damage.^18^^,^^26^ What is unique to the SJS/TEN population is the increased risk of postrenal short- and long-term effects where both males and females can experience urogenital complications such as ureteral adhesions, stenosis, or scarring leading to problems with urinary and kidney health.^18^^,^^26^

Regardless of the increased odds of experiencing pneumonia, unplanned intubations, systemic sepsis, and renal failure, we did not find any significant association with patients experiencing multiple organ failure or increased mortality in the general SJS/TEN population. We further did not observe previously described extended LOS or increased ICU admissions, but instead the opposite with shorter LOS and less likelihood of admitting an patient with SJS/TEN to an ICU when compared to their patient counterparts with burn injuries. This is likely secondary to the fact that a majority of our general SJS/TEN group consists of SJS, and our control group includes patients with burn injuries being cared for at burn centers whom are also acutely ill and require higher levels of care thus not exposing a difference in acuity.^3^^,^^4^^,^^21^^,^^31^^,^^32^

Our study’s strength lies in the size of the cohort over a decade’s time and the ability to compare to a substantive control population of patients with burn injuries cared for in similar settings across the United States. We controlled for important confounders when looking at patients with respiratory complications when controlling for inhalation injury in our comparable burn population. Nevertheless, this study is not without its limitations. Our primary and secondary outcomes were limited by the data given to us by a large national registry composed of burn centers throughout the United States with their own practice patterns. Within our study, we combined all separate pathologies of SJS, SJS-TEN overlap, and TEN as a single entity (SJS/TEN) as part of our study to include more individuals within our analysis as many of these individuals were missing TBSA to be included in our subcohort analysis. Finally, we were unable to assess the causative agents (ie, medications vs infection) preceding the diagnosis of SJS/TEN or the possible cause for patients short-term complications including reasons for intubation.

## CONCLUSION

By examining the factors such as demographic characteristics, comorbidities, and early complications in the first and largest multi-institutional analysis using the ABA NBRD, we were able to identify that elderly, housed, Black or Asian females suffering from SJS/TEN are predominantly cared for in burn centers throughout the United States when compared to the general burn population. Patients with SJS-TEN overlap and TEN experience a higher likelihood of unplanned intubations, pneumonia, sepsis, and renal failure during their stay in general with many other complications such as respiratory failure, renal failure, multiple organ failure, and mortality with increasing TBSA involvement. In general, patients with SJS/TEN cared for in burn centers are less likely to experience death, respiratory failure, need for ICU level of care, and have shorter LOS than the general burn population cared for in similar settings. In the future, we look to collaboration of all parties involved in care of patient with SJS/TEN, including burn surgeons, dermatologists, pulmonologists, gynecologists, and infectious disease doctors to reach a consensus on patient management knowing more about the population and complications which they experience to help optimize care and improve survival and recovery.

## Notes

Author contributions: Tyler J. Murphy (Conceptualization [equal], Data curation [lead], Formal analysis [equal], Investigation, Writing—original draft, Writing—review & editing [lead]), Arman J. Fijany (Conceptualization, Data curation, Formal analysis [equal], Writing—original draft, Writing—review & editing [supporting]), Emily P. Swafford (Conceptualization, Data curation, Formal analysis [equal], Writing—original draft, Writing—review & editing [supporting]), Jordan T. Garcia (Conceptualization, Data curation [equal], Writing—review & editing [supporting]), Punit Vyas (Conceptualization, Data curation, Formal analysis [equal], Writing—original draft, Writing—review & editing [supporting]), Robel T. Beyene (Conceptualization, Data curation, Formal analysis [supporting], Writing—original draft, Writing—review & editing [equal]), Stephen P. Gondek (Conceptualization, Data curation, Formal analysis [equal], Writing—review & editing [supporting]), Anne L. Wagner (Conceptualization, Data curation, Formal analysis, Writing—original draft, Writing—review & editing [equal]), Mayur B. Patel (Supervision, Writing—original draft, Writing—review & editing [equal]), and Elizabeth Dale Slater (Conceptualization, Data curation, Formal analysis [supporting], Supervision [lead], Writing—original draft, Writing—review & editing [supporting])

Funding: Drs Murphy and Patel were supported in part by the National Institutes of Health under award Number T32GM135094.

## References

[1] Lyell A . Toxic epidermal necrolysis: an eruption resembling scalding of the skin. *Br J Dermatol*. 1956;68:355–361. 10.1111/j.1365-2133.1956.tb12766.x 13374196

[2] Zimmerman D, Dang NH. Stevens–Johnson syndrome (SJS) and toxic epidermal necrolysis (TEN): immunologic reactions. In: Nates JL, Price KJ (eds.), *Oncologic Critical Care*, pp. 267–280. Springer International Publishing, 2020.

[3] Hsu DY, Brieva J, Silverberg NB, Silverberg JI. Morbidity and mortality of Stevens-Johnson syndrome and toxic epidermal necrolysis in United States adults. *J Invest Dermatol*. 2016;136:1387–1397. 10.1016/j.jid.2016.03.023 27039263

[4] Charlton OA, Harris V, Phan K, Mewton E, Jackson C, Cooper A. Toxic epidermal necrolysis and Steven-Johnson syndrome: a comprehensive review. *Adv Wound Care*. 2020;9:426–439. 10.1089/wound.2019.0977 PMC7307670 32520664

[5] Bastuji-Garin S, Rzany B, Stern RS, Shear NH, Naldi L, Roujeau JC. Clinical classification of cases of toxic epidermal necrolysis, Stevens-Johnson syndrome, and erythema multiforme. *Arch Dermatol*. 1993;129:92–96. 8420497

[6] Bettuzzi T, Lebrun-Vignes B, Ingen-Housz-Oro S, Sbidian E. Incidence, in-hospital and long-term mortality, and sequelae of epidermal necrolysis in adults. *JAMA Dermatol*. 2024;160:1288–1296. 10.1001/jamadermatol.2024.3575 39356525 PMC11447629

[7] Fathima S, Grainge MJ, Wainman H, Swiderski M, Gran S. Risk factors for the development of Stevens–Johnson syndrome/toxic epidermal necrolysis following drug administration: a systematic review and meta-analysis. *Clin Exp Dermatol*. 2024;49:1699–1704. 10.1093/ced/llae183 38747398

[8] Bastuji-Garin S, Fouchard N, Bertocchi M, Roujeau JC, Revuz J, Wolkenstein P. SCORTEN: a severity-of-illness score for toxic epidermal necrolysis. *J Invest Dermatol*. 2000;115:149–153. 10.1046/j.1523-1747.2000.00061.x 10951229

[9] Ubukata N, Nakatani E, Hashizume H, Sasaki H, Miyachi Y. Risk factors and drugs that trigger the onset of Stevens–Johnson syndrome and toxic epidermal necrolysis: a population-based cohort study using the Shizuoka Kokuho database. *JAAD Int*. 2023;11:24–32. 10.1016/j.jdin.2022.12.002 36818677 PMC9932121

[10] Chi MH, Chung WH, Hui RCY, et al. Clinical features and outcomes in children with Stevens-Johnson syndrome and toxic epidermal necrolysis. *J Dermatol*. 2022;49:895–902. 10.1111/1346-8138.16476 35715971

[11] Mockenhaupt M, Viboud C, Dunant A, et al. Stevens-Johnson syndrome and toxic epidermal necrolysis: assessment of medication risks with emphasis on recently marketed drugs. The EuroSCAR-study. *J Invest Dermatol*. 2008;128:35–44. 10.1038/sj.jid.5701033 17805350

[12] Frantz R, Huang S, Are A, Motaparthi K. Stevens-Johnson syndrome and toxic epidermal necrolysis: a review of diagnosis and management. *Med Kaunas Lith*. 2021;57:895. 10.3390/medicina57090895 PMC8472007 34577817

[13] Palmieri TL, Greenhalgh DG, Saffle JR, et al. A multicenter review of toxic epidermal necrolysis treated in U.S. burn centers at the end of the twentieth century. *J Burn Care Rehabil*. 2002;23:87–96. 10.1097/00004630-200203000-00004 11882797

[14] Shanbhag SS, Chodosh J, Fathy C, Goverman J, Mitchell C, Saeed HN. Multidisciplinary care in Stevens-Johnson syndrome. *Ther Adv Chronic Dis*. 2020;11:2040622319894469. 10.1177/2040622319894469 32523661 PMC7236394

[15] Brogan TV . Epidermal necrolyis: a skin disease to take your breath away. *Crit Care Med*. 2014;42:210–211. 10.1097/CCM.0b013e3182a51f06 24346531

[16] Hung CC, Liu WC, Kuo MC, Lee CH, Hwang SJ, Chen HC. Acute renal failure and its risk factors in Stevens-Johnson syndrome and toxic epidermal necrolysis. *Am J Nephrol*. 2009;29:633–638. 10.1159/000195632 19155617

[17] Revuz J, Penso D, Roujeau JC, et al. Toxic epidermal necrolysis. Clinical findings and prognosis factors in 87 patients. *Arch Dermatol*. 1987;123:1160–1165. 10.1001/archderm.123.9.1160 3632000

[18] Jacobsen A, Olabi B, Langley A, et al. Systemic interventions for treatment of Stevens-Johnson syndrome (SJS), toxic epidermal necrolysis (TEN), and SJS/TEN overlap syndrome. *Cochrane Database Syst Rev*. 2022;3:CD013130. 10.1002/14651858.CD013130.pub2 35274741 PMC8915395

[19] Lee HY, Walsh SA, Creamer D. Long-term complications of Stevens-Johnson syndrome/toxic epidermal necrolysis (SJS/TEN): the spectrum of chronic problems in patients who survive an episode of SJS/TEN necessitates multidisciplinary follow-up. *Br J Dermatol*. 2017;177:924–935. 10.1111/bjd.15360 28144971

[20] Houschyar KS, Tapking C, Borrelli MR, et al. Stevens-Johnson syndrome and toxic epidermal necrolysis: a systematic review and meta-analysis. *J Wound Care*. 2021;30:1012–1019. 10.12968/jowc.2021.30.12.1012 34881995

[21] American Burn Association . National Burn Repository. Published online 2023. https://www.ameriburn.org/burn-care-team/research/research-dataset

[22] Zimmermann S, Sekula P, Venhoff M, et al. Systemic immunomodulating therapies for Stevens-Johnson syndrome and toxic epidermal necrolysis: a systematic review and meta-analysis. *JAMA Dermatol*. 2017;153:514–522. 10.1001/jamadermatol.2016.5668 28329382 PMC5817620

[23] Gregory MS, Faunce DE, Duffner LA, Kovacs EJ. Gender difference in cell-mediated immunity after thermal injury is mediated, in part, by elevated levels of interleukin-6. *J Leukoc Biol*. 2000;67:319–326. 10.1002/jlb.67.3.319 10733091

[24] Jeschke MG, Van Baar ME, Choudhry MA, Chung KK, Gibran NS, Logsetty S. Burn injury. *Nat Rev Dis Primer*. 2020;6:11. 10.1038/s41572-020-0145-5 PMC7224101 32054846

[25] Saeed H, Mantagos IS, Chodosh J. Complications of Stevens-Johnson syndrome beyond the eye and skin. *Burns J Int Soc Burn Inj*. 2016;42:20–27. 10.1016/j.burns.2015.03.012 25865527

[26] Marks ME, Botta RK, Abe R, et al. Updates in SJS/TEN: collaboration, innovation, and community. *Front Med*. 2023;10:1213889. 10.3389/fmed.2023.1213889 PMC10600400 37901413

[27] Lebargy F, Wolkenstein P, Gisselbrecht M, et al. Pulmonary complications in toxic epidermal necrolysis: a prospective clinical study. *Intensive Care Med*. 1997;23:1237–1244. 10.1007/s001340050492 9470079 PMC7095164

[28] Williams R, Hodge J, Ingram W. Indications for intubation and early tracheostomy in patients with Stevens-Johnson syndrome and toxic epidermal necrolysis. *Am J Surg*. 2016;211:684–688.e1. 10.1016/j.amjsurg.2015.12.011 26860621

[29] Duong TA, de Prost N, Ingen-Housz-Oro S, et al. Stevens-Johnson syndrome and toxic epidermal necrolysis: follow-up of pulmonary function after remission. *Br J Dermatol*. 2015;172:400–405. 10.1111/bjd.13505 25496398

[30] Holm C, Hörbrand F, von Donnersmarck GH, Mühlbauer W. Acute renal failure in severely burned patients. *Burns J Int Soc Burn Inj*. 1999;25:171–178. 10.1016/s0305-4179(98)00144-2 10208394

[31] Yang MS, Lee JY, Kim J, et al. Incidence of Stevens-Johnson syndrome and toxic epidermal necrolysis: a nationwide population-based study using National Health Insurance Database in Korea. *PLoS One*. 2016;11:e0165933. 10.1371/journal.pone.0165933 27835661 PMC5106005

[32] Sekula P, Dunant A, Mockenhaupt M, et al. Comprehensive survival analysis of a cohort of patients with Stevens-Johnson syndrome and toxic epidermal necrolysis. *J Invest Dermatol*. 2013;133:1197–1204. 10.1038/jid.2012.510 23389396

